# Fungal keratitis caused by a dematiaceous Pleosporales member following ocular trauma with a wooden fragment: a case report

**DOI:** 10.1186/s12348-026-00620-y

**Published:** 2026-07-09

**Authors:** Masayuki Inuzuka, Kiyofumi Mochizuki, Takashi Yaguchi, Katsuhiko Kamei, Hirokazu Sakaguchi, Toshio Hisatomi

**Affiliations:** 1 Department of Ophthalmology, Graduate School of Medicine, Gifu University, Gifu, Japan; 2 Medical Mycology Research Center, Chiba University, Chiba, Japan; 3 Department of Ophthalmology and Visual Science, Graduate School of Biomedical and Health Sciences, Hiroshima University, Hiroshima, Japan

**Keywords:** Fungal keratitis, Pleosporales, Dematiaceous fungi, Molecular identification, Antifungal treatment

## Abstract

**Background:**

Fungal keratitis is a vision-threatening infection. Keratitis caused by dematiaceous fungi belonging to or related to the order Pleosporales is rare and diagnostically challenging because culture growth may be slow, morphology may be nonsporulating, and species-level identification may require molecular methods.

**Case presentation:**

A 44-year-old male forestry worker developed right ocular pain and a corneal ulcer after ocular trauma with a wooden fragment. He was referred to our hospital approximately one month after the injury; this first visit was defined as Day 0 (202X/11/27). At presentation, best-corrected visual acuity was 1.0 in the right eye. Slit-lamp examination showed a localized gray-white stromal infiltrate at the temporal peripheral cornea in the 9–10 o’clock position, with irregular margins and an overlying fluorescein-positive epithelial defect, but without hypopyon. The ocular media were sufficiently clear for detailed fundus examination, and dilated fundus examination showed no clinical evidence of posterior segment involvement or endophthalmitis. B-scan ultrasonography was therefore not performed. Direct microscopy using 10% potassium hydroxide wet mount and Fungiflora Y staining, corneal culture, and a 12-strip/24-target multiplex real-time polymerase chain reaction panel were all negative at Day 0. After temporary cessation of antifungal therapy because of severe drug-induced hepatotoxicity during systemic voriconazole and acetazolamide therapy (aspartate aminotransferase 540 U/L, alanine aminotransferase 1041 U/L), the lesion enlarged. Repeat scraping culture yielded a filamentous fungus on Day 58. The isolate formed black, slightly velvety colonies and dark hyphae, but did not produce diagnostic conidia or spores under the conditions used. Sequencing of the ribosomal DNA internal transcribed spacer region by Sanger sequencing, followed by BLAST comparison, suggested that the isolate belonged to the order Pleosporales, although species-level identification was not achieved. Treatment consisted of stepwise combination therapy, including topical voriconazole, amphotericin B, and natamycin, initial systemic voriconazole, subsequent topical amphotericin B, systemic and topical micafungin, and oral terbinafine. After active infection had clinically stabilized, 20% autologous serum eye drops were added to support epithelial healing. At the final follow-up on Day 268, best-corrected visual acuity was 1.2, with a quiet anterior segment and a stable corneal scar.

**Conclusions:**

This case highlights the importance of repeated microbiological sampling and molecular identification in traumatic fungal keratitis when initial tests are negative. It also illustrates the need for cautious, individualized treatment when azole therapy is interrupted because of systemic toxicity. Because species-level identification and antifungal susceptibility testing were not achieved, the therapeutic implications of this single case should be interpreted conservatively.

## Background

Fungal keratitis is a vision-threatening form of infectious keratitis and remains an important cause of corneal blindness worldwide [1]. A wide variety of filamentous fungi and yeasts, including *Candida*, *Fusarium*, and *Aspergillus*, can cause this condition. Management is often difficult because diagnosis may be delayed, antifungal susceptibility varies among organisms, and topical antifungal agents have limited availability and penetration [1].

In Japan, culture-positive fungal keratitis includes both yeast-like and filamentous fungi. In a multicenter prospective observational study, 94 fungal strains were isolated from 93 cases, including 42 yeast-like fungi and 52 filamentous fungi; *Fusarium* spp. were the most frequent filamentous fungi, whereas *Candida albicans* and *Candida parapsilosis* were the most frequent yeast-like fungi [2]. Thus, both ocular-surface/post-surgical contexts and trauma-associated filamentous fungal infection should be considered in Japan. In the same Japanese multicenter framework, combination-drug susceptibility testing showed that the in vitro effects of antifungal combinations can differ according to the strain and drug pairing [3].

Pleosporales is a large order of dematiaceous fungi that includes plant-associated and environmental species [4]. Human infection caused by Pleosporales members is uncommon, but deep cutaneous infections and rare ocular or nail infections due to related taxa have been reported [5, 6]. In traumatic keratitis after exposure to plant or woody material, such rare dematiaceous fungi may be considered when routine tests are negative and culture morphology is non-diagnostic.

Rare keratitis cases caused by dematiaceous fungi such as *Cladosporium* spp. and *Stemphylium* spp. have been reported after trauma [7, 8]. Because these organisms may grow slowly and may not produce diagnostic conidia or spores in culture, species-level identification based on morphology alone can be difficult. ITS sequencing and database comparison can support higher-level molecular identification, although additional loci such as RPB2 may be required for more reliable species-level identification [4, 9, 10].

Antifungal susceptibility among dematiaceous fungi varies by species, and MIC data are often unavailable for rare isolates [11, 12]. Accordingly, therapeutic conclusions from single cases should be interpreted cautiously.

Here, we report a case of fungal keratitis caused by a dematiaceous Pleosporales member following ocular trauma by a wooden fragment in a forestry worker. Initial direct microscopy, culture, and a 12-strip/24-target multiplex real-time PCR panel were negative, and pathogen identification was delayed. Severe drug-induced hepatotoxicity during systemic voriconazole and acetazolamide therapy necessitated major modification of treatment. We describe the clinical course, culture characteristics, and ITS-based molecular identification, and discuss diagnostic and therapeutic considerations in the context of fungal keratitis in Japan.

### Case presentation

#### Patient information

The patient was first examined at our hospital on 202X/11/27; this presentation date is defined as Day 0 throughout the report.

A 44-year-old man presented with right ocular pain. His past medical, family, and allergy histories were unremarkable. He had no underlying conditions such as diabetes mellitus or collagen vascular disease and no history of immunosuppressive therapy. He worked in forestry and was routinely exposed to trees and wood fragments.

While at work, he sustained blunt trauma to the right eye from a wooden fragment and visited a local clinic shortly thereafter. He was diagnosed with traumatic iritis and treated with topical corticosteroids. However, ocular pain persisted, and approximately one month after the injury, a corneal ulcer developed in the right eye. Corneal scraping at the referring clinic revealed a fungal organism, but the lesion responded poorly to both topical and systemic antifungal therapy. He was therefore referred to our hospital for further evaluation and management.

### Ophthalmic evaluation

At the initial visit, best-corrected visual acuity (BCVA), intraocular pressure (IOP), slit-lamp biomicroscopy, anterior segment photography, and dilated fundus examination were performed. Slit-lamp examination assessed the location and size of the corneal lesion, the characteristics of stromal infiltration, the extent of epithelial defect, and the presence or absence of anterior chamber inflammation, including hypopyon. Fluorescein staining was used to evaluate the relationship between the epithelial defect and stromal infiltration. Posterior segment involvement, including a possible endophthalmitis component, was evaluated clinically by dilated fundus examination because the ocular media were sufficiently clear. B-scan ultrasonography was not performed. The fellow eye was examined in the same manner.

**Fig. 1:**
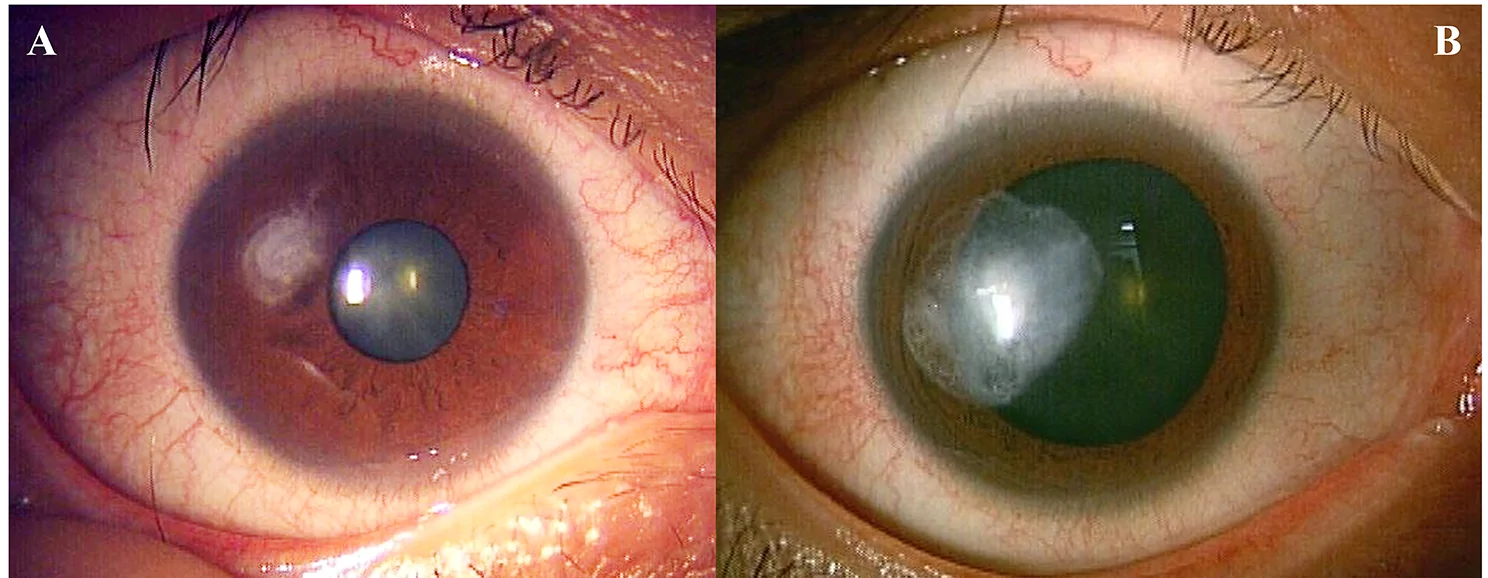
Slit-lamp photographs of the right eye. (**A**) At presentation to our hospital (Day 0): a localized gray-white stromal infiltrate with irregular margins and an overlying epithelial defect is present in the temporal peripheral cornea at the 9–10 o’clock position. No hypopyon is observed. (**B**) Day 268: the cornea shows stable scarring with near-complete resolution of active inflammation

At presentation, BCVA was 1.0 in the right eye and 1.5 in the left eye. IOP measured by non-contact tonometry was 20.5 mmHg in the right eye and 14.0 mmHg in the left eye. Slit-lamp examination of the right eye revealed a localized gray-white stromal infiltrate in the temporal peripheral cornea at the 9–10 o’clock position. The infiltrate had irregular, partly feathery margins with mild surrounding stromal haze, and fluorescein staining demonstrated an overlying epithelial defect that approximately corresponded to the infiltrated area (Fig. 1A). No obvious satellite lesions, endothelial plaque, hypopyon, or marked anterior chamber inflammation was observed. The ocular media were sufficiently clear to allow detailed fundus examination. Dilated fundus examination showed no vitreous opacity, retinal infiltrates, retinal detachment, chorioretinal lesions, or other findings suggestive of posterior segment involvement or endophthalmitis. Therefore, B-scan ultrasonography was not performed. The left eye showed no abnormal findings.

### Microbiological examinations

Corneal scrapings were collected from both the margin and base of the ulcer using a sterile spatula. The specimen was divided: one portion was used for potassium hydroxide (KOH) wet-mount microscopy and fluorescent staining with Fungiflora Y, and the remaining portion was submitted for culture. Direct microscopy with KOH wet mount and Fungiflora Y staining at Day 0 was negative.

Cultures were performed on multiple media, including blood agar and Sabouraud agar, and incubated at 30 °C and 35 °C for up to four weeks. All cultures were monitored for bacterial and fungal growth. When a filamentous fungus was isolated, it was subcultured on potato dextrose agar (PDA) for morphological assessment. At the initial visit, a 12-strip/24-target multiplex real-time PCR panel was also performed to evaluate bacteria, viruses, and major fungal species. When disease progression or recurrence was suspected during follow-up, corneal scraping was repeated and submitted for additional culture and molecular analyses.

**Fig. 2:**
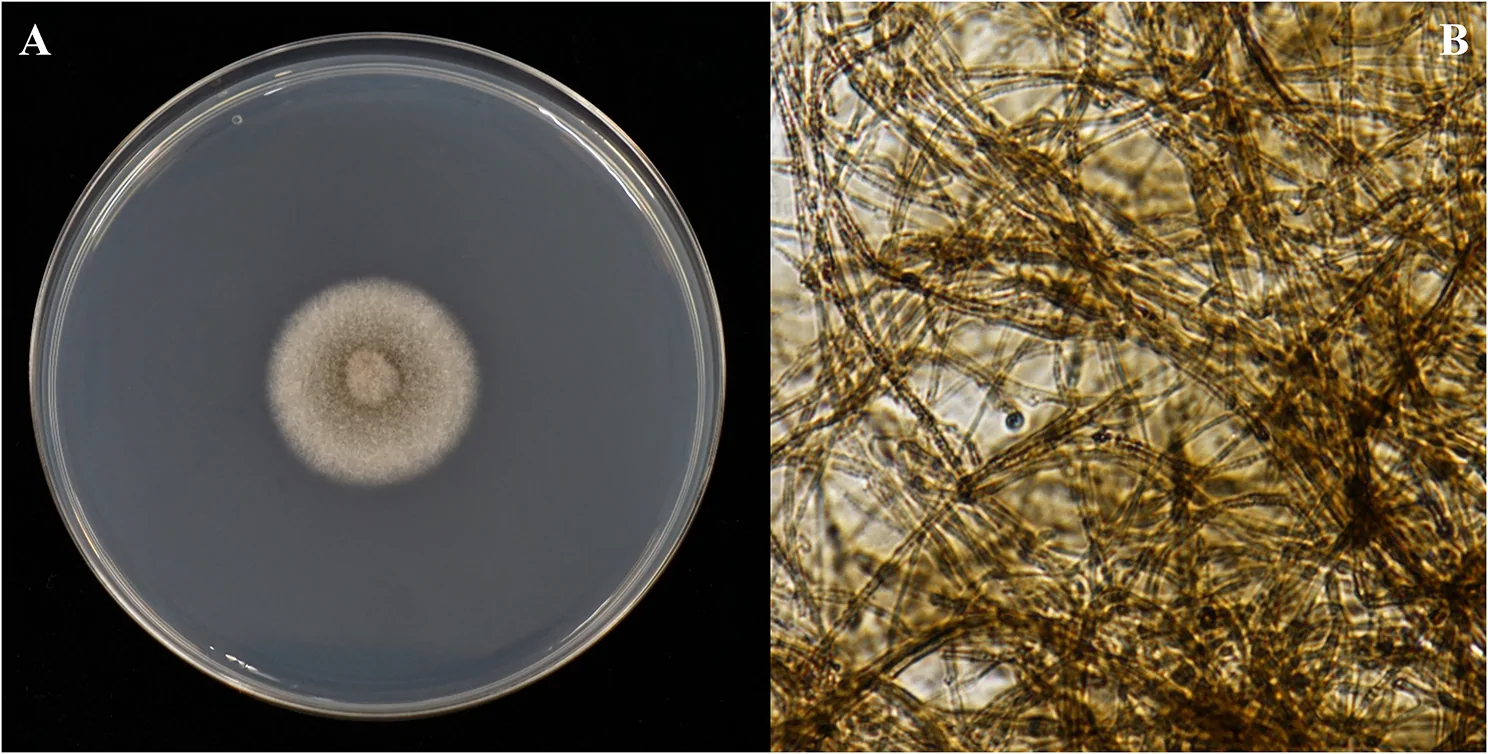
Colony morphology of the isolated fungus. (**A**) Colony appearance on potato dextrose agar after 7 days of incubation at 25°C, showing black, slightly velvety colonies. (**B**) Microscopic appearance showing dark hyphae without diagnostic conidia or spores under the conditions used

The initial corneal scraping culture was negative for both bacteria and fungi. The 12-strip/24-target multiplex real-time PCR panel was also completely negative. In contrast, corneal scraping performed at the referring clinic had demonstrated an unidentified fungal organism. After temporary interruption of treatment because of drug-induced hepatotoxicity, the corneal lesion enlarged, prompting repeat corneal scraping on Day 51 for culture and molecular analyses. On Day 58, filamentous fungal growth was detected for the first time at our hospital, confirming recurrent fungal keratitis. On PDA after 7 days of incubation at 25 °C, the isolate formed black, slightly velvety colonies. Microscopic examination showed dark hyphae, but no diagnostic conidia, sexual structures, or other spores were produced under the conditions used, precluding reliable genus- or species-level identification based on morphology alone (Fig. 2).

#### Molecular identification

Genomic DNA was extracted from the filamentous fungal isolate obtained from culture. Polymerase chain reaction (PCR) targeting the ribosomal DNA internal transcribed spacer (ITS) region was performed, and the amplified products were sequenced using the Sanger method. The obtained ITS sequence was compared with publicly available sequences of Dothideomycetes and Pleosporales fungi registered in the NCBI database using BLAST. Molecular identification was therefore based on ITS sequencing and BLAST comparison.

BLAST analysis revealed high similarity to several dematiaceous fungi belonging to the order Pleosporales, but no complete match with any known species. These findings suggested that the pathogen belonged to the order Pleosporales; however, definitive species-level identification was not achieved.

The isolate was preserved as IFM 67,273 at the Medical Mycology Research Center, Chiba University through the National Bio-Resource Project, Japan (JPMBRP-202228). The ITS sequence data are available from the corresponding author upon reasonable request.

### Therapeutic interventions and clinical course

From Day 0 of treatment at our hospital, 1% voriconazole eye drops and 0.1% amphotericin B eye drops were administered hourly. Natamycin eye drops four times daily and natamycin eye ointment three times daily were also prescribed. Systemic voriconazole was started at 400 mg/day. Cefmenoxime and atropine eye drops were added. Because of elevated IOP, oral acetazolamide was initiated at 400 mg/day on Day 16 and increased to 600 mg/day on Day 29.

Although the corneal lesion initially showed slight improvement, severe hepatotoxicity developed on Day 39 while the patient was receiving systemic voriconazole and acetazolamide (aspartate aminotransferase 540 U/L, alanine aminotransferase 1041 U/L). After consultation with physicians in the Department of Internal Medicine, immediate discontinuation of all potentially causative medications, including topical and systemic antifungal agents and oral acetazolamide, was recommended because drug-induced liver injury was marked and the responsible medication could not be determined with certainty. Therefore, all antifungal agents and oral acetazolamide were temporarily discontinued. During this period, only artificial tears and atropine eye drops, which were considered to have minimal systemic hepatic impact and were required for supportive ocular management, were continued. We recognize that temporary cessation of topical antifungal therapy was not ideal from the perspective of infectious keratitis management; however, it was undertaken as a patient-safety measure under guidance from physicians in the Department of Internal Medicine in response to marked hepatotoxicity. In retrospect, earlier repeat microbiological sampling and earlier reintroduction of non-azole topical antifungal therapy could have been considered once the systemic condition had improved.

As the corneal lesion subsequently enlarged, natamycin eye ointment was restarted on Day 51, and corneal scraping was repeated for culture and PCR. Repeat corneal scraping was selected because the lesion remained clinically localized to the cornea and because no clinical evidence of posterior segment involvement or endophthalmitis was present on dilated fundus examination. More invasive procedures, such as endoscraping, corneal biopsy, or anterior chamber tap, were not performed. After filamentous fungal growth was detected on Day 58 and liver function had improved, 0.1% amphotericin B eye drops were resumed, and intravenous micafungin was initiated at 600 mg per dose, administered three times weekly in an outpatient setting due to the patient’s scheduling constraints.

Clinical improvement remained slow and prolonged. Oral loxoprofen was added on Day 65, and weekly corneal scrape cultures were performed to monitor disease activity. Because improvement of the corneal lesion remained insufficient, 0.1% micafungin eye drops were started on Day 128 and administered four times daily, and oral terbinafine 125 mg/day was added on Day 137.

After active infection had clinically stabilized but delayed epithelial recovery persisted, 20% autologous serum eye drops were prepared aseptically, diluted with normal saline, and administered four times daily from Day 191 to support epithelial healing. Thereafter, conjunctival hyperemia and subjective symptoms gradually improved. By Day 226, both systemic and ocular conditions had markedly improved. At the final visit on Day 268, BCVA was maintained at 1.2, and the cornea showed stable scarring without active inflammation (Fig. 1B).

### Discussion and conclusions

This case describes traumatic fungal keratitis caused by a dematiaceous fungus that was molecularly classified within the order Pleosporales in an immunocompetent forestry worker. The clinically important features were diagnostic difficulty after initial negative testing, nonsporulating dark fungal colonies that could not be identified by morphology alone, treatment modification because of severe drug-induced hepatotoxicity, and a favorable visual outcome after prolonged stepwise management.

Initial KOH wet mount, Fungiflora Y staining, culture, and multiplex real-time PCR were negative at our hospital, despite prior fungal detection at the referring clinic. Possible reasons include limited sample volume, low fungal burden at the time of sampling, prior antifungal exposure, slow growth of dematiaceous fungi, and the fact that the multiplex panel targeted common ocular pathogens rather than rare Pleosporales members. The lesion enlarged after the medically directed treatment interruption, and repeat scraping subsequently yielded the causative filamentous fungus. The prolonged cessation of topical antifungal treatment may have contributed to lesion enlargement and to discrepancies between the clinical course and microbiological findings. However, the interruption occurred in the context of marked drug-induced liver injury, uncertainty regarding the responsible medication, and recommendation by physicians in the Department of Internal Medicine to discontinue potentially causative medications. This course supports repeated sampling when clinical suspicion remains high, particularly after plant- or wood-related trauma. Earlier repeat scraping, endoscraping, corneal biopsy, or anterior chamber tap could have been considered while maintaining or reintroducing topical non-azole antifungal coverage as soon as clinically feasible.

The isolate produced black, slightly velvety colonies and dark hyphae but did not produce diagnostic spores. Pleosporales is morphologically diverse, and culture characteristics can vary depending on growth conditions [4, 10]. In the present case, ITS sequencing and BLAST comparison supported classification within the order Pleosporales, but not species-level identification. Because no phylogenetic tree or multi-locus analysis was performed, the taxonomic interpretation was deliberately restricted to order-level classification.

Therapeutic interpretation should be cautious. Pleosporales and other dematiaceous fungi show heterogeneous antifungal susceptibility patterns, and MIC data were not available for this isolate [11, 12]. Systemic voriconazole had to be discontinued because severe hepatotoxicity developed during treatment with systemic voriconazole and acetazolamide. We cannot determine which subsequent component of therapy—topical amphotericin B, systemic or topical micafungin, oral terbinafine, repeated debridement/scraping, or natural reduction of fungal load—was primarily responsible for disease control. The favorable outcome should be regarded as the result of individualized stepwise management rather than evidence for a specific non-azole regimen.

The use of autologous serum eye drops in this case was supportive rather than antifungal. They were introduced late in the clinical course, after active infection had clinically stabilized, because prolonged inflammation and intensive topical antifungal treatment were associated with delayed epithelial recovery. Autologous serum contains epitheliotrophic factors, including growth factors, fibronectin, and vitamins, and has been used for severe ocular surface disease and persistent corneal epithelial defects [13, 14]. In the present case, autologous serum was used to promote epithelial healing and improve ocular surface stability, not to treat the fungal infection directly.

This case is clinically relevant in Japan because the epidemiology of fungal keratitis includes both yeast-like and filamentous organisms [2]. Although many Japanese cases occur in ocular-surface or post-surgical contexts, this case occurred after wood-fragment trauma in a forestry worker, a setting in which rare plant- or soil-associated filamentous fungi should be considered.

Several limitations must be acknowledged. First, this is a single case, and the findings cannot be generalized to all Pleosporales-associated keratitis. Second, species-level identification was not achieved. Molecular identification was based on ITS sequencing and BLAST comparison only. A phylogenetic tree and multi-locus phylogenetic analysis, including genes such as RPB2, were not performed. Third, antifungal susceptibility testing was not available, limiting conclusions about optimal therapy. Fourth, because multiple treatments were sequentially or concurrently used, the independent effect of each drug and the supportive effect of autologous serum cannot be determined. Fifth, the temporary interruption of antifungal therapy may have influenced the clinical course and microbiological findings; this interruption was made under guidance from physicians in the Department of Internal Medicine because of marked drug-induced hepatotoxicity, but earlier repeat sampling and earlier reintroduction of topical non-azole antifungal therapy could have been considered. Finally, the initial negative PCR result should not be interpreted as evidence that multiplex PCR is ineffective for fungal keratitis; rather, it reflects the limitations of targeted panels, sample volume, and fungal burden in this particular case.

In conclusion, rare dematiaceous fungi within the order Pleosporales should be considered in traumatic keratitis after exposure to wood or plant material, even when initial direct microscopy, culture, and multiplex PCR are negative. Repeated corneal scraping and molecular identification can reduce diagnostic delay and improve etiological classification. When antifungal treatment must be interrupted because of systemic toxicity, treatment should be modified in close collaboration with physicians in relevant specialties while recognizing the importance of reintroducing appropriate topical antifungal coverage as soon as safely feasible. Further accumulation of molecularly confirmed cases with susceptibility testing and multi-locus analysis is needed to clarify the clinical spectrum and optimal management of Pleosporales-associated keratitis.

## Data Availability

All data generated or analysed during this study are included in this published article. Additional details are available from the corresponding author upon reasonable request.

## Abbreviations

BCVA: Best-corrected visual acuity
BLAST: Basic Local Alignment Search Tool
IOP: Intraocular pressure
ITS: Internal transcribed spacer
KOH: Potassium hydroxide
MIC: Minimum inhibitory concentration
PCR: Polymerase chain reaction
PDA: Potato dextrose agar
